# Three phylogenetically distinct and culturable diazotrophs are perennial symbionts of leaf‐cutting ants

**DOI:** 10.1002/ece3.8213

**Published:** 2021-12-14

**Authors:** Renata de Oliveira Aquino Zani, Milene Ferro, Maurício Bacci

**Affiliations:** ^1^ Centro de Estudos de Insetos Sociais (CEIS) Universidade Estadual Paulista (UNESP) Rio Claro ‐ SP Brazil; ^2^ Departamento de Biologia Geral e Aplicada Universidade Estadual Paulista (UNESP) Rio Claro ‐ SP Brazil

**Keywords:** 16S rRNA, Attini, *nif*H, nitrogen fixation, nutritional symbiosis

## Abstract

The obligate mutualistic basidiomycete fungus, *Leucocoprinus gongylophorus*, mediates nutrition of leaf‐cutting ants with carbons from vegetal matter. In addition, diazotrophic Enterobacteriales in the fungus garden and intestinal Rhizobiales supposedly mediate assimilation of atmospheric nitrogen, and Entomoplasmatales in the genus *Mesoplasma*, as well as other yet unidentified strains, supposedly mediate ant assimilation of other compounds from vegetal matter, such as citrate, fructose, and amino acids. Together, these nutritional partners would support the production of high yields of leafcutter biomass. In the present investigation, we propose that three phylogenetically distinct and culturable diazotrophs in the genera *Ralstonia*, *Methylobacterium,* and *Pseudomonas* integrate this symbiotic nutrition network, facilitating ant nutrition on nitrogen. Strains in these genera were often isolated and directly sequenced in 16S rRNA libraries from the ant abdomen, together with the nondiazotrophs *Acinetobacter* and *Brachybacterium*. These five isolates were underrepresented in libraries, suggesting that none of them is dominant *in vivo*. Libraries have been dominated by four uncultured Rhizobiales strains in the genera *Liberibacter*, *Terasakiella,* and *Bartonella* and, only in *Acromyrmex* ants, by the Entomoplasmatales in the genus *Mesoplasma*. *Acromyrmex* also presented small amounts of two other uncultured Entomoplasmatales strains, *Entomoplasma* and *Spiroplasma*. The absence of Entomoplasmatales in *Atta* workers implicates that the association with these bacteria is not mandatory for ant biomass production. Most of the strains that we detected in South American ants were genetically similar with strains previously described in association with leafcutters from Central and North America, indicating wide geographic dispersion, and suggesting fixed ecological services.

## Graphical abstract

In leaf‐cutting ant pests, an obligate mutualistic basidiomycete mediates the ants’ nutrition with carbons from vegetal matter using a fermenter‐like system inside the nest, called a “fungus garden.” Diazotrophs such as Enterobacteriales in the fungus garden and intestinal Rhizobiales were proposed to mediate the incorporation of atmospheric nitrogen into ant proteins; Entomoplasmatales were proposed to power the fungus metabolism. Leafcutters likely rely on a microbial community to access the carbon and nitrogen necessary for the production of the millions of individuals found in a single giant colony. In the present investigation, we found that mutualism between the leafcutter and Rhizobiales occurs in a wide geographic range and mainly between three closely related species within the Rhizobiales genera Liberibacter, Bartonella, and Terasakiella. However, the association between the ant and the Entomoplasmatales genus Mesoplasma is likely parasitic or commensal, since giant leafcutter colonies may be either massively invaded by or completely devoid of these bacteria, suggesting that these endosymbionts are not essential for high yields of ant biomass. We also found three new candidate mutualists, frequently isolated from the ant abdomen, including nearly identical diazotrophs of the genera Methylobacterium (Rhizobiales) and Ralstonia (Burkholderiales) and nondiazotrophs of the genus Acinetobacter. These results point to multiple noncompetitive and complementary strategies for atmospheric nitrogen nutrition within the leafcutter system.

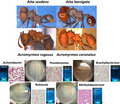

## 1 INTRODUCTION

Leaf‐cutting ants, that is, those in the *Atta*, *Acromyrmex,* and *Amoimyrmex* genera, are endemic in a vast geographic area in North, Central, and South America (Cristiano et al., 2020; Hölldobler & Wilson, 1990). These characteristics, as well as their specialized behavior, social organization, and pest status, have made them excellent study models (Rabeling et al., 2008).

The ecological success of leafcutters relies on their associations with microorganisms. The primary association occurs with a basidiomycete fungus, *Leucocoprinus gongylophorus*, also referred to as *Leucoagaricus gongylophorus*, which the ants cultivate on leaf material inside the nests, in a fermenter‐like system, called a “fungus garden,” which mediates the assimilation of carbon by ants from vegetal polysaccharides (Mueller et al., 2017; Quinlan & Cherrett, 1979; Silva et al., 2003; Somera et al., 2015).

In addition to the mutualistic fungus, diazotrophic bacteria may also contribute to the ant diet, potentially mediating the assimilation of atmospheric nitrogen; examples are *Klebsiella* and *Pantoea* isolates (Enterobacteriales) living in the fungus garden (Pinto‐Tomás et al., 2009) and uncultured Rhizobiales inhabiting the ants’ intestines (Sapountzis et al., 2015; Zhukova et al., 2017).

Uncultured Entomoplasmatales may also contribute to ants’ diet. Representatives within the genus *Mesoplasma* (Mollicutes) have frequently been found in ant samples from the United States and Brazil (Meirelles et al., 2016), including the *Mesoplasma* OTU 1544. This strain is nearly identical to EntAcro1 retrieved from the guts of Panamanian leafcutters (Sapountzis et al., 2015) and proposed to supplement ants’ diet with acetate to provide extra energetic input, supporting the larger biomass production by leafcutters (Sapountzis et al., 2018). Another Entomoplasmatales strain, EntAcro10, has also been proposed to facilitate ant nutrition on simple sugars and amino acids (Sapountzis et al., 2018). However, the relative amounts of *Mesoplasma* associated with ants can be as low as 0.14%, so the proposed nutritional benefits could be comparably small in these cases, and amounts of *Mesoplasma* over 80% were excessively high and proposed to be related to the death of leaf‐cutting ant colonies (Meirelles et al., 2016).

This controversy illustrates the challenges of characterizing the relationship between leafcutters and nutritional bacteria. Other reasons contributing to that include incomplete biogeographic information, poor cultivability of microbial symbionts in laboratory conditions, and high diversity and variability of bacteria groups living integrated in the ant environment (Barcoto et al., 2020; Ronque et al., 2020). Therefore, the current scenario suggests that multiple bacterial associations may be related to the ants, so it is conceivable that other, yet noncharacterized, nutritional players exist.

Aiming to add more information concerning the ubiquity, geographic distribution and role of bacteria participating in the symbiosis with leaf‐cutting ants, we sampled colonies from São Paulo state in Brazil and identified and compared the bacteria associated with the sanitized and nonsanitized ant abdomen, by means of high‐throughput DNA sequencing. We found that Rhizobiales were always the dominant group, consistently with a perennial symbiotic relationship; however, Entomoplasmatales in the genus *Mesoplasma* may either dominate or be absent from the giant and healthy ant colonies, consistently with an occasional and nonobligate symbiotic association. In addition, the Rhodobacterales genus *Rhodovulum* was often but not always found in relatively large numbers.

**FIGURE 1:**
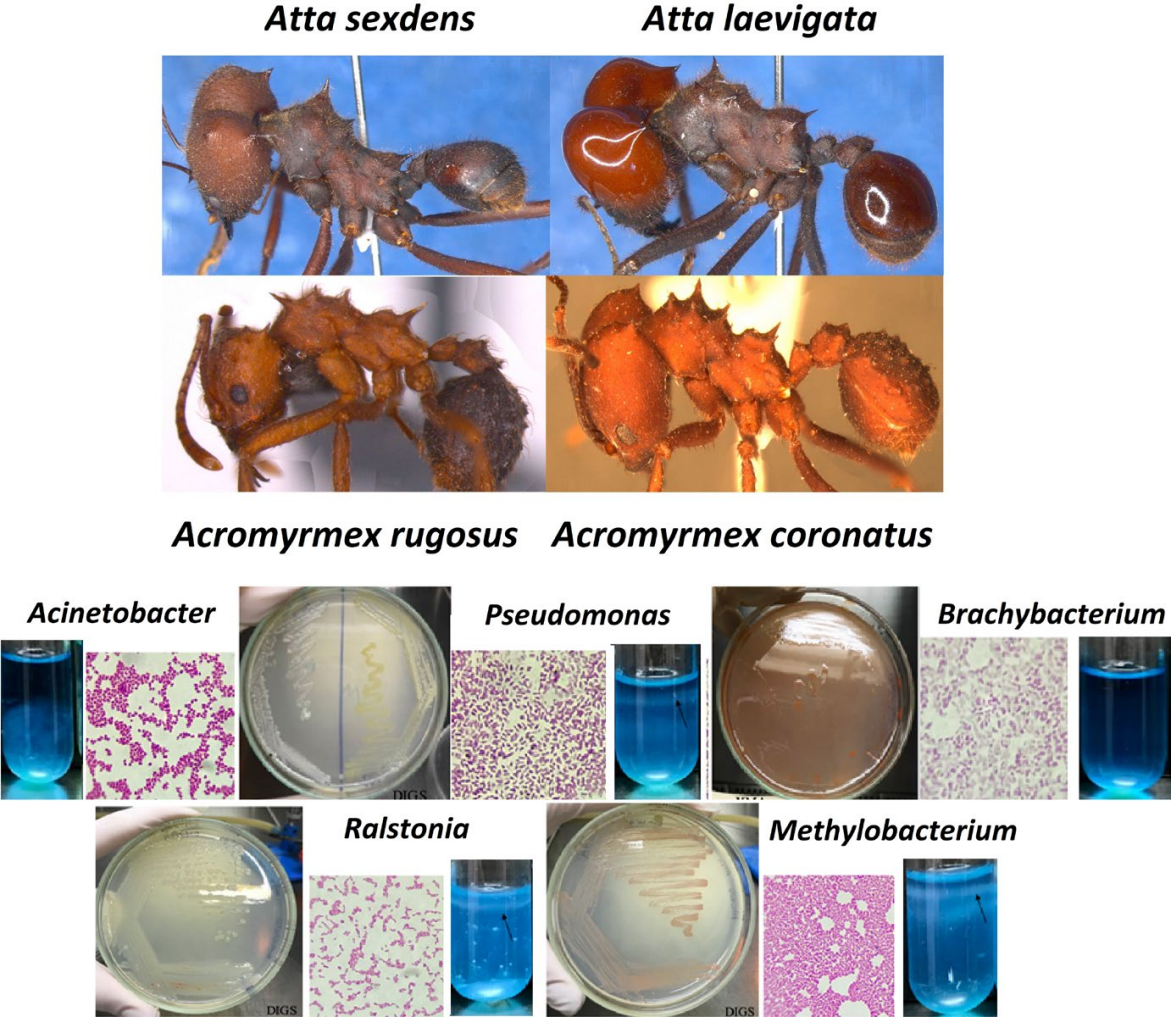
Leafcutter ant species sampled, and main bacteria strains isolated from ant abdomen. Isolated bacterial strains were cultured in Petri dishes and observed under the microscope after Gram staining. The formation of a growth film in NFb culture medium (arrows) indicated the ability to assimilate atmospheric nitrogen by *Pseudomonas*, *Ralstonia*, and *Methylobacterium* isolates

We also followed four leafcutter ant colonies for 24 months, periodically isolating bacterial representatives from the ant abdomen, and consistently found nearly identical diazotrophs of the *Ralstonia*, *Methylobacterium,* and *Pseudomonas* genera and nondiazotrophs of the genera *Acinetobacter* and *Brachybacterium* (Figure 1). These isolates likely tightly adhere to the ant's body and the diazotrophs are new candidates for nutritional symbionts, possibly associated with nitrogen fixation and recycling for leaf‐cutting ants. Because these are culturable strains, biochemical and physiological tests will be facilitated in the future, which may provide new insights in the study of symbiotic relationships between ants and microbes.

## 2 METHODS

### 2.1 Collection and identification of ants

We sampled soldiers from five ant colonies in the counties of Rio Claro and Itirapina, these sites being 40 km distant from each other, in southeastern Brazil: 10–12 mm long *Atta laevigata* individuals from either Rio Claro (22°23′42″S, 47°32′33″W) or Itirapina (22°14′41″S, 47°50′38″W), 8–11 mm *Atta sexdens* individuals from Rio Claro (22°23′44″S, 47°32′47″W), 6–8 mm *Acromyrmex rugosus* from Rio Claro (22°23′42″S, 47°32′44″W), and 6–8 mm *Acromyrmex coronatus* from Rio Claro (22°23′43″S, 47°32′32″W). Itirapina colony was used for microbial isolation procedures for checking the occurrence of isolates in a different geographic site as compared to Rio Claro colonies. Rio Claro colonies were used for assessing microbial diversity in a single geographic region but in four different leafcutter species, as well as molecular studies. Samples were generally collected at the entrance of the nest; *Acromyrmex coronatus* samples were collected on the trail due to the difficulty of accessing the arboreal nest. The species were identified using barcode sequencing of mitochondrial genes COI and COII (Hebert et al., 2003) and then using differentiation keys (Borgmeier, 1959; Gonçalves, 1961). To extract DNA, individual specimens were crushed using a mortar and pestle containing 250 µl TNES (0.25 M Tris–HCl; 2 M NaCl; 0.1 M EDTA; 2.5% SDS pH 7.5). The samples were transferred for 1.5 microtubes; then, 10 μl of 20 mg/ml proteinase K was added to the microtubes and the mixture was incubated for 3 h at 55°C, followed by addition of 5 µl of 10 mg/ml RNAse and 30 min incubation at 37°C. Proteins were then precipitated by adding 200 µl of 5 M NaCl, the supernatant was collected, and DNA was precipitated with 100% isopropanol, washed with 70% ethanol, centrifuged, dried, and dissolved in 50 µl pH 8.0 TE buffer. PCR amplification of mitochondrial COI and COII genes was conducted using 10 µM primers ANT‐F (ATTCATTCTTATCTTGAAATATTATTTC), and ANT‐R (TTCATAAGTTCAGTATCATTGGTG) was used for PCR amplification with PCR Master Mix (GeneDireX. Inc.; Martins et al., 2007). The integrity and purity of samples were observed on 1.0% agarose gel, and Amplicon concentration was measured using a NanoDrop 2000 spectrophotometer (Thermo Scientific) and purified using exonuclease I and alkaline phosphatase enzymes (Thermo Fisher Scientific). Sequencing was performed by applying the same forward or reverse primers used for amplification and using the 0.5 μl BigDye™ terminator sequencing kit version 3.1 (Applied Biosystems™). These reactions included a 95°C incubation for 1 min, followed by 28 cycles of 20 s at 95°C, 40 s at 50°C, and 4 min at 60°C. The products of the sequencing reaction were purified and then resolved on a Sanger Sequencing 3500 Series Genetic Analyzer (Applied Biosystems^®^). The generated sequences were edited with the BIOEDIT 7.0.5 program (Hall, 1999), followed by BLASTn analysis (v.2.6.0+; Altschul et al., 1990) using the GenBank NR database as a reference.

### 2.2 Sanitization of ant samples

Before DNA extraction and sequencing, the ants were sequentially washed once with 5 ml 70% ethanol, once with 5 ml of 2% sodium hypochlorite, and six times with 8 ml of ultrapure water. Single large *Atta* soldiers or pairs of smaller *Acromyrmex* soldiers were placed in a single tube and then were gently homogenized manually by inversion for 10 s in each tube. The purpose of sanitization was to remove microorganisms weakly adhered to the ant cuticle.

### 2.3 DNA extraction, 16S rRNA library preparation and sequencing, read preprocessing and analysis

Eight libraries were prepared, two for each of the Rio Claro colonies, either before or after sanitizing the ant samples. The ant abdomen from 2 to 4 ant soldiers was removed and macerated into a fine powder in liquid nitrogen using a mortar and pestle; then, approximately 20 mg (wet weight) was subjected to DNA extraction with the Cells and Tissue DNA isolation kit (Product # 53100) from Norgen Biotek Corp according to the manufacturer's instructions. The extracted DNA was sent to Macrogen Inc., where amplicon libraries of the V3–V4 region of the 16S rRNA gene were prepared, multiplexed, submitted to next‐generation sequencing (NGS) on the MiSeq Illumina platform (paired‐end 2× 300 bp), and demultiplexed. We then performed quality control in the FastQC program version 0.11.5 (Babraham Bioinformatics; www.bioinformatics.babraham.ac.uk/projects/fastqc) to search for adapters and primers and verify sequence quality per base. We found no adapter or primer sequences and trimmed out low‐quality raw reads (Phred Q score <20) using SeqyClean (Zhbannikov et al., 2017) and carried out taxonomic identification with the OmicsBox platform (BioBam Bioinformatics S.L., Valencia, Spain) using the taxonomic sequence classification system Kraken (Wood & Salzberg, 2014), which applied exact search and phylogenetic methods to compare each obtained sequence with the curated Kraken database version 2019_06, with high performance, sensitivity, and accuracy (Lindgreen et al., 2016; Lu & Salzberg, 2020). Rarefaction curves were generated in the OmicsBox platform and represented as the number of expected bacterial genera (*Y*‐axis) per number of reads (*X*‐axis). Principal coordinates analysis (PCoA) was implemented using OmicsBox with the Bray–Curtis distances to compare bacterial community composition in different libraries. Bacterial alpha‐diversity indices were estimated on the same platform.

### 2.4 Bacterial genera shared within ant species

Venn diagrams were generated with Bioinformatics & Evolutionary Genomics tools (http://bioinformatics.psb.ugent.be/webtools/venn/), to identify bacterial genera that were shared between ant colony samples.

### 2.5 Bacterial isolation and characterization

In total, 23 isolation experiments were performed on the five studied ant colonies from October 2017 to September 2019. *Atta* soldiers are larger than their *Acromyrmex* counterparts; therefore, fewer *Atta* were collected (10) compared with *Acromyrmex* (15) from each sampling. Each soldier was washed, the abdomen was separated and macerated with a pestle in a 1.5‐ml Eppendorf tube, and portions of the macerate were streaked on solid culture media surfaces. Washing removed many of the fast‐growing yeasts, making bacterial isolation more efficient. For the isolation of Rhizobiales, the culture medium used was YMA (yeast extract mannitol agar, pH 7.0) from Sigma‐Aldrich (cod. 101417911) and 0.25% Congo red (Sigma‐Aldrich; cod. 6767). The media were incubated for approximately 3–4 days at 30°C and colonies were shown stained in red (Fred & Waksman, 1928). The nutrient‐rich DIGS (Table S4) medium facilitates bacterial growth in relatively small incubation time and was used for the isolation of slow‐growing diazotrophic bacterial species, with incubation at 30°C for 3–4 days (Döbereiner et al., 1999; Sabino et al., 2012). Culture media are described in Table S4. Isolates were stained with the Gram protocol (Holt et al., 1994) and inspected under the microscope.

### 2.6 Molecular identification of bacterial isolates

DNA extraction was performed as described by Sampaio et al. (2001) with some modifications. Two loopfuls of each isolate were suspended in 500 µL of lysis solution (Tris 50 mmol/L, NaCl 250 mmol/L, EDTA 50 mmol/L, and SDS 0.3% at pH 8.0), glass beads were added, and the suspension was vortexed for 4 min and incubated in a dry bath at 65°C for 1 h, and centrifuged at 18,000 *g* for 15 min. The aqueous phase was transferred to a 1.5‐ml microtube and stored at −20°C. Amplification was performed using 10 µM of 16S rRNA universal primers 27F (AGAGTTTGATCCTGGCTCAG) and 1492R (GGTTACCTTGTTACGACTT; Lane, 1991). PCRs contained 9 µl of ultrapure water, 9 µl of 2× PCR Master Mix (GeneDireX Inc.), 0.5 µl of each primer solution, and 1 µl of DNA sample. PCR conditions included an initial 95°C incubation for 3 min followed by 30 cycles of 95°C for 30 s, 55°C for 30 s, and 72°C for 90 s, followed by a final extension of 72°C for 5 min. Amplicon purification and sequencing followed the procedures described in Section 2.1. The generated sequences were preprocessed with the BIOEDIT 7.0.5 program (Hall, 1999), and the taxonomic assignments of each sequence were performed using the Ribosomal Database Project classifier (Cole et al., 2009) and Kraken (Wood & Salzberg, 2014) in the OmicsBox platform. Sequence percent identity was calculated using multiple alignments in the MAFFT program (Katoh et al., 2002) or local Blastn pairwise alignment (Altschul et al., 1990).

### 2.7 PCR amplification of the *nif*H gene

Amplification was performed using 10 µM of primers 19F (GCIWTYTAYGGIAARGGIGG), 407R (AAICCRCCRCAIACIACRTC) conditions described by Ueda et al. (1995), with some modifications, changing the annealing temperature. Amplification was carried out with a final PCR volume of 20 µl, 9 µl of ultrapure water, 9 µl of 2× PCR Master Mix (GeneDireX. Inc.), 0.5 µl of each primer, and 1 µl of DNA. PCR conditions included an initial 94°C incubation for 3 min followed by 40 cycles of 94°C for 30 s, 46°C for 1 min, and 72°C for 1 min, followed by a final extension of 72°C for 3 min. We used the nitrogen fixative *Klebsiella pneumonia* (Roberts et al., 1978) as the positive control. Amplicon purification and sequencing were performed following the procedures described in Section 2.1. The generated sequences were edited using the BIOEDIT 7.0.5 program (Hall, 1999) and identified using the BLASTn v.2.6.0 program (Altschul et al., 1990) against the NR database.

### 2.8 Analysis of nitrogen fixation

Isolates were subjected to physiological analysis for nitrogen fixation using *Klebsiella pneumoniae* as the positive control. The test was performed in tubes containing 5 ml of semi‐solid, nitrogen‐free NFb culture medium for approximately 72 h at 28°C. This assay was performed in duplicate. The formation of a growth film in the culture medium indicated the capacity to fix nitrogen (Döbereiner et al., 1995). After film formation, isolates were streaked in Petri dishes containing YMA and molecularly identified. Culture media composition is shown in Table S4.

### 2.9 Comparison between isolated colonies and 16S rRNA libraries

We created a formatted local database BLAST for each 16S rRNA library and used the OmicsBox platform to run a BLASTn search (E‐value cutoff 1e‐03) against Sanger sequences obtained from every isolate. When no hit was found, the isolate was considered absent from the 16S rRNA library. If hits were found, identities were calculated using Blastn for the first 500 best hits. We also mapped reads from each of the 16S rRNA libraries to the Sanger 16S rRNA sequences, using the Bowtie2 (Langmead & Salzberg, 2012) tool to verify the number of reads corresponding to each of the isolates.

### 2.10 Comparison of 16S rRNA libraries with 16S rRNA sequences from previously described leafcutter symbionts

The five bacterial genera individually representing 26%–93% of 16S rRNA library sequences (Table 1) were regarded as dominant genera. For each dominant genus, we selected a representative sequence that was assembled using CAP3 (Huang & Madan, 1999). BLASTn (minimum E‐value 1e‐03) in the OmicsBox platform was used to compare each of these sequences against GenBank ones already described for bacteria associated with leafcutters, such as Rhizobiales (RhiAcro1, GenBank accession # KR336619), *Mesoplasma* (EntAcro1—GenBank accession # KR336618), and *Entomoplasma* (EntAcro2, GenBank accession # KR336617) obtained from *Acromyrmex* (Sapountzis et al., 2015). We also compared each representative sequence with those kindly provided by Lucas Meirelles (Meirelles et al., 2016).

**TABLE 1:** Bacteria identified in the leafcutter abdomen by 16S rRNA sequencing or isolation methods

Next‐generation sequencing					Isolation			
Sample (colony number)	16S rRNA library read pairs/classified	Shannon (H′)	Simpson (C)	16S rRNA library order/genus (% reads)	Order/genus	*nif*H	Growth on N_‐_free medium	Found in the 16S rRNA library [identity (read %)] #
*Atta laevigata* Sanitized (Rio Claro)	108,938/99.87%	2.724	0.8502	Rhizobiale*s*/*Liberibacter* (88.58) Rhizobiales/*Terasakiella* (1.3) Rhodobacterales/*Rhodovulum* (0.5)	Rhizobiales/*Methylobacterium* Burkholderiales/*Ralstonia* Actinomycetales/*Brachybacterium* Pseudomonadales/*Acinetobacter*	+ + − −	−/+ + − −	86.7–94.2 (7.2) No 83.8–93.6 (0.006) 94.7–98.6 (0.017)
*Atta laevigata* (Rio Claro)	126,678/99.77%	3.688	0.9406	Rhizobiales/*Liberibacter* (89.45) Rhodobacterales/*Rhodovulum* (0.78)				
*Atta sexdens* Sanitized (Rio Claro)	82,680/99.9%	3.016	0.8298	Rhizobiales/*Bartonella* (92.98) Rhodobacterales/*Rhodovulum* (1.48) Rhizobiales/*Liberibacter* (1.17)	Burkholderiales/*Ralstonia* Rhizobiales/*Methylobacterium* Pseudomonadales/*Acinetobacter*	+ + −	+ + −	91.5–93.9 (0.008) 94.7–99.1 (0.59) No
*Atta sexdens* (Rio Claro)	124,222/99.73%	3.263	0.8532	Rhizobiales/*Bartonella* (80.3) Rhodobacterales/*Rhodovulum* (1.56) Rhizobiales/*Liberibacter* (1.1)				
*Acromyrmex rugosus* Sanitized (Rio Claro)	106,078/99.97%	0.84	0.4828	Entomoplasmatale*s*/*Mesoplasma* (56.42) Rhizobiales/*Terasakiella* (27.56) Entomoplasmatale*s*/*Entomoplasma* (5.84) Rhizobiales/*Liberibacter* (3.2) Rhodobacterales/*Rhodovulum* (2.27) Entomoplasmatale*s*/*Spiroplasma* (1.15)	Pseudomonadales/*Acinetobacter* Pseudomonadales/*Pseudomonas*	− +	− −/+	No No
*Acromyrmex rugosus* (Rio Claro)	119,161/99.98%	1.249	0.5944	Entomoplasmatales/*Mesoplasma* (33.73) Rhizobiales/*Terasakiella* (32.57) Rhizobiales/*Bartonella* (9.66) Rhizobiales/*Liberibacter (*8.76) Entomoplasmatales/*Entomoplasma* (3.34) Rhodobacterales/*Rhodovulum* (2.33) Entomoplasmatales/*Spiroplasma* (1.11)				
*Acromyrmex coronatus* Sanitized (Rio Claro)	100,217/99.97%	0.916	0.4868	Rhizobiales/*Terasakiella* (56.21) Entomoplasmatales/*Mesoplasma* (25.72) Rhizobiales/*Liberibacter* (5.69) Entomoplasmatales/*Entomoplasma* (3.02) Rhodobacterales/*Rhodovulum* (2.4) Entomoplasmatales/*Spiroplasma* (0.96)	Pseudomonadales/*Acinetobacter* Burkholderiales/*Ralstonia*	− +	− +	92.9–93.3 (0.11) No
*Acromyrmex coronatus* (Rio Claro)	98,703/99.96%	0.948	0.5223	Entomoplasmatale*s*/*Mesoplasma* (50.93) Rhizobiales/*Terasakiella* (31.81) Entomoplasmatales/*Entomoplasma* (5.35) Rhizobiales/*Liberibacter* (3.49) Rhodobacterales/*Rhodovulum* (2.36) Entomoplasmatales/*Spiroplasma* (1.41)				
*Atta laevigata* Sanitized (Itirapina)					Rhizobiales/*Methylobacterium* Burkholderiales/*Ralstonia* Pseudomonadales/*Acinetobacter*	+ + −	−/+ + −	

Note

# Read %: percentage of library reads mapped on a given 16S rRNA Sanger read as obtained by Bowtie2.

Representative sequences were also assigned to a bacterial genus using the Kraken in the OmicsBox platform.

## 3 RESULTS

### 3.1 Species identification

Ant species were identified using taxonomic keys and were confirmed by comparing the COI‐IGS‐tRNA‐Leu‐COII fragment (Martins et al., 2007) with sequences contained in GenBank. This comparison resulted in the closest matches with the following identities and E‐values: *Atta laevigata* from Itirapina: 92.95%/8e‐82; *Atta laevigata* from Rio Claro: 98.17/0.0; *Atta sexdens*: 99.8/0.0; *Acromyrmex rugosus*: 79.55/1e‐28; and *Acromyrmex coronatus* 88.16/7e‐20.

### 3.2 Bacterial community is species‐specific in leafcutters

**FIGURE 2:**
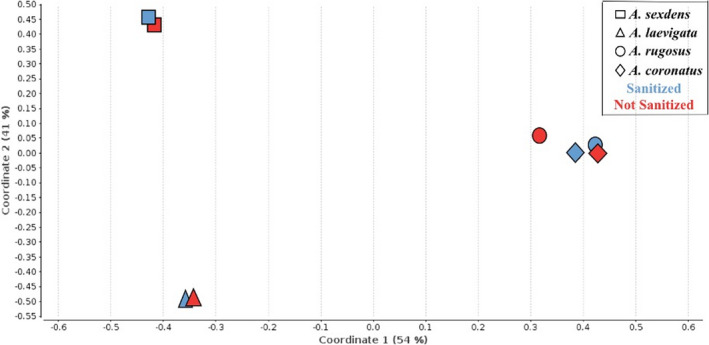
Rarefaction curves used to indicate the bacterial variety of each ant sample. The *y*‐axis shows the distinct bacterial genus, and the *x*‐axis shows the number of sequences

**FIGURE 3:**
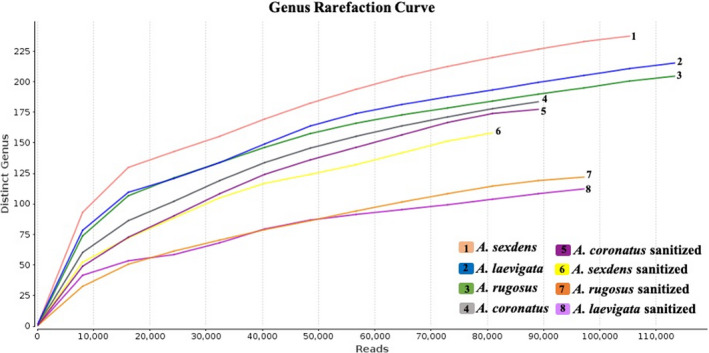
Principal coordinates analysis (PCoA) of 16S rRNA sequences for sanitized (blue) and nonsanitized (red) ant species

The 16S rRNA libraries contained 1,735,340 reads with 300 bp; after filtering, there remained 1,691,678 reads with 300 bp; after filtering, there remained 1,691,678 reads with Phred values ≥20. The gradual flattening of the rarefaction curves (Figure 2) indicates the sampling of most of the sequence diversity contained in libraries, allowing a comprehensive biodiversity characterization of bacterial communities (Wood & Salzberg, 2014). PCoA analysis (Figure 3) showed that the bacterial communities are similar in both *Acromyrmex* species, but distinct from those associated with *Atta* species; that the bacterial community associated with *Atta sexdens* is distinct from that associated with *Atta laevigata*; and that the predominant bacterial communities are similar in sanitized and nonsanitized samples of the same ant species. Shannon and Simpson indices (Table 1) showed that species of *Atta* had greater bacterial diversity than *Acromyrmex* species, and that sanitizing samples resulted in decreased diversity.

### 3.3 Four bacterial genera dominate leafcutters libraries, and three are perennial

**FIGURE 4:**
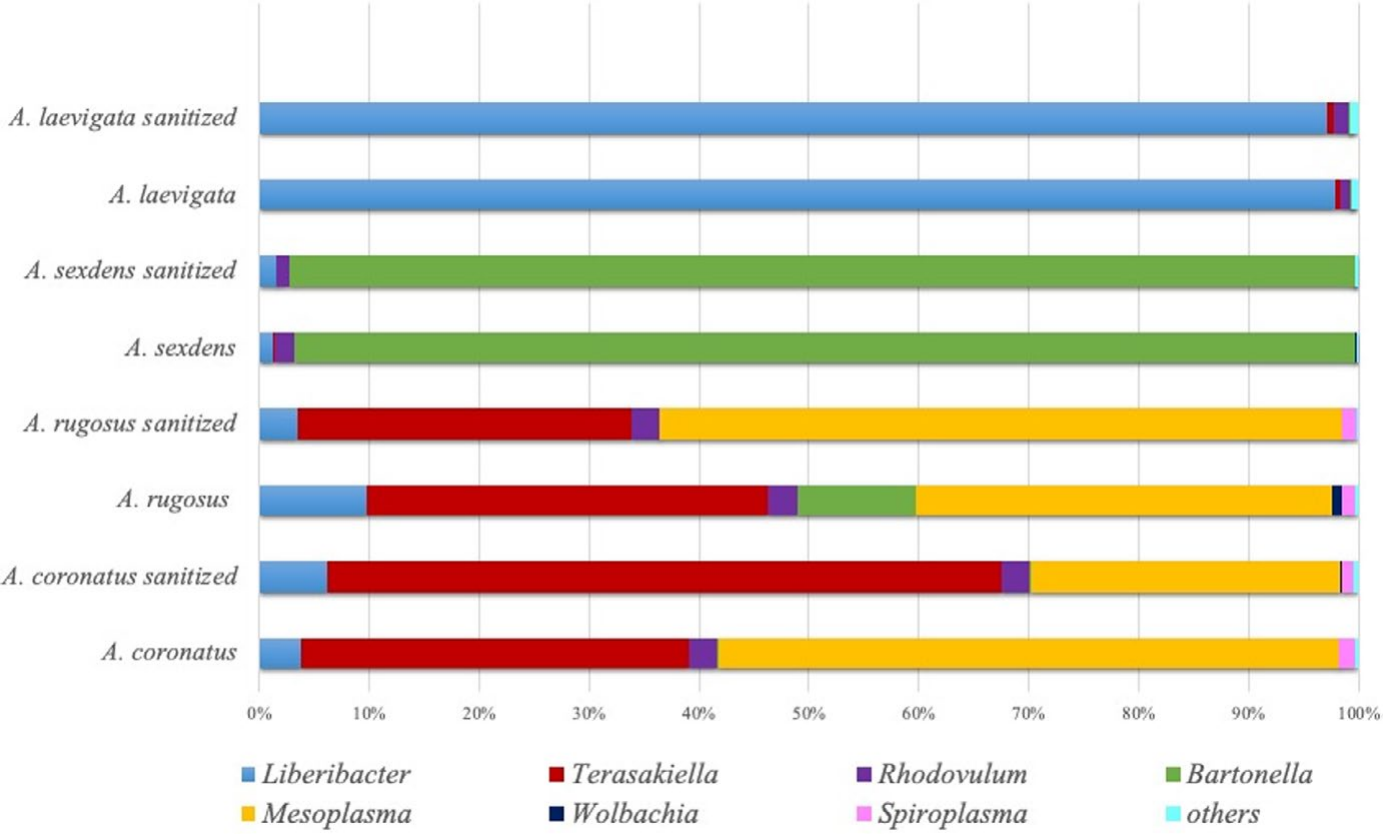
Taxonomic classification of 16S rRNA gene sequences (genus level). The legend shows the main genera obtained for each sanitized and nonsanitized ant library

**FIGURE 5:**
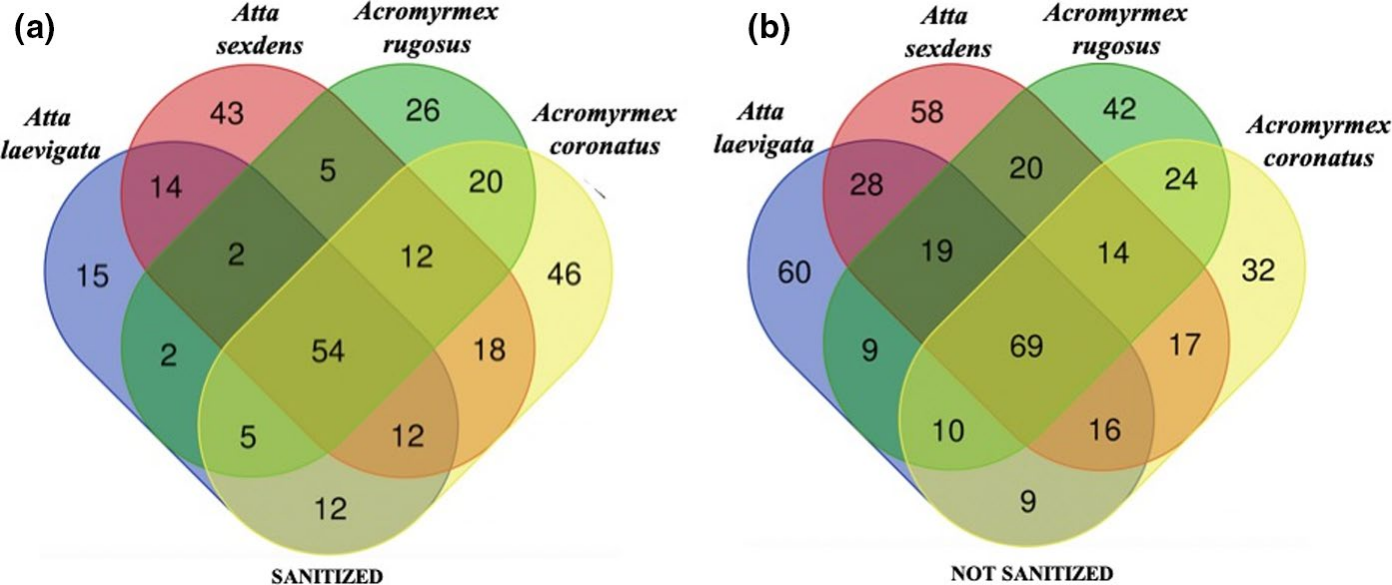
Venn diagrams for sanitized and nonsanitized ant samples showing shared or exclusive bacterial genera distribution. *Liberibacter*, *Terasakiella*, *Bartonella* and *Rhodovulum* belonging to the 54 shared genera by sanitized samples (a); *Liberibacter*, *Bartonella*, *Terasakiella*, *Rhodovulum,* and *Wolbachia* belonging to the 69 shared genera in nonsanitized samples (b)

The Kraken program successfully assigned to 502 bacterial genera 87 to 98% of the 821,541 sequences contained in our libraries. Microbial communities of each ant sample predominantly contained bacterial strains showing 16S rRNA sequences 98.6–98.9 identical to each other in three closely related specific Rhizobiales genera: *Liberibacter* in *Atta laevigata*, *Bartonella* in *Atta sexdens*, and *Terasakiella* in both *Acromyrmex rugosus* and *Acromyrmex coronatus* (Table 1 and Figure 4). Together, these three genera corresponded to 95% of the sequences shared between all libraries (Tables S1 and S2), which encompassed 54 shared bacterial genera in sanitized samples and 69 shared genera in nonsanitized samples (Figure 5).

In addition, both *Acromyrmex* species also contained significant amounts of the Entomoplasmatales *Mesoplasma*, but no sequences in this genus were detected in *Atta* species (Table 1 and Figure 4). Therefore, *Mesoplasma* was regarded as occasional and *Liberibacter*, *Bartonella* and *Terasakiella* genera as perennial. Sequences in two of these bacterial genera were successfully assigned to the species level: *Terasakiella* sp. SH‐1 and *Mesoplasma lactucae*.

### 3.4 Isolates in five genera were detected and three were able to fix nitrogen

Diazotrophic bacteria have been reported to live in association with the attine ant abdomen (Sapountzis et al., 2015) and fungus garden (Pinto‐Tomás et al., 2009); therefore, we used appropriate culture media to isolate bacterial diazotrophs from the leafcutter's abdomen. This isolation was possible after sanitization of soldiers, which removed yeasts and molds. During the two‐year period, we visited the five nests studied in the current report, collected ant samples, performed 23 isolation experiments, and obtained 42 bacterial isolates (Table S3).

Twenty‐one representative isolates were sequenced and classified in five different bacterial genera. Of these 21 isolates, 12 were found to fix nitrogen as they had the *nif*H gene and grew on N‐free media (Table 1): four nearly identical (97.4%–100% 16S rRNA) *Methylobacterium* (Order Rhizobiales) isolates from two *Atta laevigata* and one *Atta sexdens* colonies; six (85.8%–99.7%) *Ralstonia* isolates (order Burkholderiales) from *Atta laevigata*, *Atta sexdens*, and *Acromyrmex coronatus*, and two nearly identical *Pseudomonas* (Order Pseudomonadales) isolates (99.3%) from *Acromyrmex rugosus*. The 85.8% identity was a relatively low value, so genus classification within *Ralstonia* may look dubious in this case. However, genus classification was confirmed by RDP with 95% confidence interval and, in addition, *Ralstonia* sequences retrieved from NCBI (NR_134150.1, NR_134149.1, NR_134148.1, NR_118984.1, NR_044040.1, NR_114126.1, NR_113.352.1, NR_043152.1, NR_040803.1, NR_025385.1, NR_025242.2, NR_025975.1, AY792011.1, AJ507103.1, AY792033.1, KC757034.1, bNR_044040.1, NR_025975.1) showed 82 to 99.6% identity to each other, so strains currently classified within *Ralstonia* show a more diverse 16S rDNA sequence.

Notably, eight non‐nitrogen‐fixing *Acinetobacter* (Order Pseudomonadales) isolates (97%–99% identity) were found in every analyzed ant colony/species and, in addition, one *Brachybacterium* (Order Actinomycetales) was found in *Atta laevigata*. *Methylobacterium*, *Ralstonia* and *Acinetobacter* strains were prevalent, since they were found, respectively, in three, four, or five out of the five nests sampled (Table 1).

The five genera were represented in minor amounts (0.0006%–7.2%) in sequences of the 16S rRNA libraries with maximal identity variation from 93% to 99.1% (Table 1); they have been found in libraries of all ant species, being regarded as perennial. Therefore, although culturable bacteria have often been recovered from ant samples, they have been underrepresented in the 16S rRNA libraries, which were dominated by sequences from uncultured strains. Another point to be highlighted is that sequences from five of the 21 isolates (Table 1) had no hits within the NGS 16S rRNA libraries, possibly because sequencing V3 and V4 regions of the 16S rRNA gene were not sufficient for complete taxonomic identification (Zhang & You, 2018).

### 3.5 Major isolated and uncultured strains in 11 genera are geographically widespread

Based on the 16S rRNA sequence, strains in the major genera that we found in Brazil have similar or nearly identical relatives in the United States and Republic of Panama (Table 2), being therefore geographically widespread.

**TABLE 2:** Main isolated or uncultured genera, their read % found in Brazilian leafcutters, and their close relatives already described as symbiotic with leafcutters from the Republic of Panama (RP) or Texas (USA)

Detection	Genus	Read %				Id (%)	Related strain	Location	Ref.
		S^+^	L	R	C				
16S rDNA library	*Liberibacter^n^*	1.25	89.05	6.14	4.6	95.55	RhiAcro1	RP	1
	*Terasakiella^n^*	0.04	0.44	30.21	44.10	98.86			
	*Bartonella^n^*	85.36	0.13	5.13	0.09	99.09			
	*Mesoplasma*	0	0	44.41	38.23	98.47/98.9	OTU‐1544	USA	2
						99.57/99.78	EntAcro1	RP RP	1
	*Entomoplasma*	0	0	4.49	4.17	91.42/91.85	EntAcro2		
Cultivation + 16S rDNA library	*Methylobacterium* ^N^	0.03	0.03	0.03	0.03	96–99	*Methylobacterium*	Brazil	3
						96–99	165 B08NCY14_206	USA	2
	*Ralstonia* ^N^	0.0005	0.0005	0.0005	0.0005	99	984 T05BL07Y10‐167		
	*Acinetobacter*	0.04	0.04	0.04	0.04	96	1262 A10FBY14 124		
						99	57 P13NE01Y14 497		
	*Pseudomonas* ^N^	0.02	0.02	0.02	0.02	92	1510 T03BL02Y10 4861		
	*Brachybacterium*	0.008	0.008	0.008	0.008	94	942 P31NE08Y14 390394		

Note

^+^S: *Atta sexdens*; L: *Atta* laevigata; R: *Acromyrmex rugosus*; C: *Acromyrmex coronatus*. 1: Sapountzis et al. (2015); 2 Meirelles et al. (2016); 3 Martinez et al. (2019). ^N^Nitrogen‐fixing; ^n^putative nitrogen‐fixing or recycling.

The representative sequences for dominant Rhizobiales genus *Liberibacter* were similar (96% identity), and representative sequences from dominant *Bartonella* and *Terasakiella* were nearly identical (~99% identity) to that of the RhiAcro1 symbiont (GenBank accession # KR336619), which was found in the guts of *Acromyrmex* ants from Gamboa, Panama (Sapountzis et al., 2015). In addition, sequences representative from the dominant *Mesoplasma* strains found in *Acromyrmex coronatus* and *Acromyrmex rugosus* were, respectively, nearly identical (~99% identity) to EntAcro1 (GenBank accession # KR336618) from Central America or *Mesoplasma lactucae* OTU‐1544 (GenBank accession # KT248001) from North America (Meirelles et al., 2016); and the nondominant *Entomoplasma* strains from *Acromyrmex* species was similar to EntAcro2 (GenBank accession # KR336617) from Central America (Sapountzis et al., 2015) and to leafcutter symbionts from Texas (Meirelles et al., 2016). Table 2 also shows that the main five cultured isolates were also similar or nearly identical (92%–99% identity) to counterpart strains symbiotic with leaf‐cutting ants from Texas.

## 4 DISCUSSION

One of the challenges in characterizing the nutritional roles of microbes in leafcutters is the high diversity of bacteria associated with these ants, which makes it difficult to determine a specific contribution of a particular bacterial strain. In the present investigation, we aimed to shed some light on this scenario, by adding new information collected from leafcutters sampled in the state of São Paulo, Brazil. We isolated some bacterial strains and generated NGS data from others that were associated with the ant abdomen. Then, the most frequent strains were identified and classified as perennial or occasional, based on occurrence in association with four leafcutter species. With this procedure, we showed that a highly diverse core leafcutter bacteriome is probably retained in the ant abdomen by structural means, and selected five cultured strains, three of which are candidate nutritional symbionts involved in nitrogen fixation and recycling. We compared our results with those already described in previous studies on leafcutters and found that these candidates are geographically widespread. In the following subsections, we discuss the implications of these findings.

### 4.1 A core bacteriome is retained in the ants by structural means

In our NGS experiments, we found 502 genera associated with leafcutters, most of them within Alphaproteobacteria, which was represent by a large proportion of the sequences described in 16S rRNA libraries, corroborating previous studies for several species of leaf‐cutting ants (Liberti et al., 2015; Sapountzis et al., 2015, 2019; Teseo et al., 2019; Vieira et al., 2017). Our community analysis (Figure 3) suggests that some leafcutter–microbe associations may be species‐specific, as described in basally derived Attini (Ronque et al., 2020). On the other hand, some associations were detected in all ant species, as represented by a core leafcutter bacteriome with 84 bacterial genera (Tables S1 and S2), which may result from fixed symbiotic associations with shared functional roles, perhaps related to ant nutrition.

As expected, after sanitizing the ants, the bacterial diversity decreased, as detected by a reduction in the average values for both Shannon (18%) and Simpson (9%) indices, as well as the average read number (15% decrease) in libraries (Table 1). This indicates that immersion of soldiers in 70% ethanol followed by 2% sodium hypochlorite and ultrapure water was not sufficient to remove most of the bacterial cells associated with the ant abdomen. However, the sanitizing procedure massively removed yeasts and molds detectable in culture media. Therefore, the resistance of removal suggests that some bacteria are retained by the ants using structural means, which may facilitate the housing of the core leafcutter bacteriome.

### 4.2 Six candidate nutritional symbionts may support nitrogen metabolism in leafcutters

The core leafcutter bacteriome contains eleven selected candidates as major symbionts. Among them, six strains in six different bacterial genera caught our attention because they may support nitrogen metabolism in ants. Three of these strains, *Methylobacterium* (Rhizobiales), *Ralstonia* (Burkholderiales), and *Pseudomonas* (Pseudomonadales) were detected in libraries of all ant species (Table 2) and are able to fix nitrogen.

*Methylobacterium* strains closely related with our isolates (Table 2) have already been found associated with the cuticle of *Acromyrmex coronatus* (Martinez et al., 2019) and with a pouch‐shaped organ located in the intestines of the ant *Tetraponera* (93%–97% similarity to our sequence), which possibly domesticated these microbes as endosymbionts that recycle nitrogen (van Borm et al., 2002).

Another main isolated strain was a diazotroph in the genus *Ralstonia* (Order Burkholderiales), obtained from *Atta sexdens*, *Atta laevigata*, and *Acromyrmex coronatus*, with 16S rRNA sequence showing 91.5%–93.9% identity to other *Ralstonia* sequences detected in *Atta sexdens* libraries (Table 1). Bacteria in the *Ralstonia* genus had already been described with no obvious function in fungal gardens of *Atta* species (Suen et al., 2010), in the refuse dumps of *Atta cephalotes* (Ortiz‐Reyes et al., 2016) and the larval intestines of *Atta cephalotes* (Zhukova et al., 2017).

Diazotrophic isolates within *Ralstonia* or *Methylobacterium* corresponded to 10 out of the 12 nitrogen‐fixing isolates consistently found over 24 months in *Atta* and *Acromyrmex* colonies, so they may have a major role for the ants. However, compared to uncultured *Liberibacter*, *Bartonella*, *Terasakiella,* or *Mesoplasma*, they were poorly detected in 16S RNA libraries, which is not compatible with a major quantitative impact on nutrient budgets (see below the discussion on collaborative ecological services provided by symbionts).

We also found *Pseudomonas* isolates in *Acromyrmex rugosus*, and bacteria in this genus have been previously isolated from the cuticle of *Acromyrmex coronatus* (Martinez et al., 2019) and molecularly described in larvae of *Acromyrmex echinatior* and *Atta cephalotes* (Zhukova et al., 2017) or adults of *Atta texana* (Meirelles et al., 2016).

The three other candidates that could support nitrogen metabolism in ants are those overrepresented in the NGS libraries, corresponding to the perennial and closely related *Liberibacter*, *Terasakiella,* and *Bartonella* (Rhizobiales). They are genetically similar with RhiAcro1, a dominant extracellular Rhizobiales strain forming an intestinal biofilm confined to the lumen of three *Acromyrmex* species, with potential to fix (Sapountzis et al., 2015) and recycle (Sapountzis et al., 2018) nitrogen. In addition, *Bartonella* strains described in *Harpegnathos saltator* (Neuvonen et al., 2016) and in the intestines of *Dolichoderus* and *Cephalotes* may be nutritional symbionts associated with nitrogen recycling (Bisch et al., 2018; Hu et al., 2018).

### 4.3 Entomoplasmatales are occasional symbionts

Gut Entomoplasmatales have been proposed to be nutritional symbionts able to supplement ants’ diet with acetate and involved in nitrogen recycling (Sapountzis et al., 2018). Our results indicate that Entomoplasmatales are not present in all leafcutters, because these microbes have been only found in our *Acromyrmex* species, *Mesoplasma* as dominant, and *Entomoplasma* and *Spiroplasma* in a smaller percentage within the 16S rRNA libraries (Table 1). These strains were not detected in four *Atta sexdens* or *Atta laevigata* libraries encompassing 865,516 16S rRNA reads and made from workers of two healthy and active colonies, which, therefore, have apparently been producing ant biomass without association with Entomoplasmatales. These findings indicate that Entomoplasmatales are occasional symbionts, which does not fit the hypothesis (Sapountzis et al., 2018) that additional energy generated by a gut *Mesoplasma* was necessary for leafcutters to reach large‐scale farming that is associated with high productivity of insect biomass; or with the hypothesis that *Mesoplasma* could be a permanent mutualist of *Atta texana* (Meirelles et al., 2016). Alternative roles for *Mesoplasma* have been suggested, like protecting Ponerinae ants from infection by other prokaryotes (de Oliveira et al., 2016) or parasitizing leafcutters (Meirelles et al., 2016).

### 4.4 Three major symbionts remain with no proposed function

Tables S1 and S2 show three other main isolates for which sparse information is available regarding association with ants. *Rhodovulum* (Rhodobacterales) is the fourth most frequent dominant in the core bacteriome, but we found no other *Rhodovulum* sequences associated with leafcutters in public databases. *Acinetobacter* isolates were found in the four analyzed ant species and detected in the 16S rRNA library of *Atta laevigata* and *Acromyrmex coronatus*. Isolates of this genus have already been reported in association with the cuticle of *Acromyrmex coronatus* (Martinez et al., 2019), intestines of *Atta cephalotes* larvae (Zhukova et al., 2017), thorax, abdomen, and head of *Atta texana* queens (Meirelles et al., 2016); and associated with ants of the genera *Polyrhachis* (Ramalho et al., 2017), *Pheidole* (Martins & Moreau, 2020); and with the digestive tract of the parasitoid wasp *Nasonia* (Brucker & Bordenstein, 2012). However, no specific function was shown for *Acinetobacter* and the same doubts remain for *Brachybacterium*, which has been occasionally reported associated with ants and plants (Chen et al., 2021; Kautz et al., 2013).

### 4.5 Main detected symbionts likely provide ecological services for leafcutters

Table 2 shows that all major uncultured microbes (six genera) and cultured ones (five genera) identified have nearly identical or very similar relatives living with leafcutters in Central or in North America. High genetic similarity indicates a wide geographic dispersion of these bacterial symbionts, which suggests fixed ecological services provided to the host ants.

Some ecological services have been already proposed, such as (1) mediation of leafcutter nutrition on atmospheric nitrogen provided by Enterobacteriales in the genera *Klebsiella* and *Pantoea* living in the fungus garden (Pinto‐Tomás et al., 2009) and (2) nitrogen assimilation and recycling by uncultured Rhizobiales inhabiting the ants’ intestines (Sapountzis et al., 2015; Zhukova et al., 2017).

Our current results add other partners, which likely contribute to leafcutter nutrition on nitrogen: culturable abdominal diazotrophs in the genera *Ralstonia* (Burkholderiales), *Methylobacterium* (Rhizobiales), and *Pseudomonas* (Pseudomonadales), so leafcutters seem to rely on multiple complementary and phylogenetically distinct nutritional partners to produce biomass on a large scale.

## CONFLICT OF INTEREST

The authors declare that they have no competing interests.

## AUTHOR CONTRIBUTION

**Renata de Oliveira Aquino Zani:** Investigation (equal); methodology (equal). **Milene Ferro:** Investigation (equal); methodology (equal). **Maurício Bacci Jr:** Resources (supporting); supervision (supporting); writing‐review and editing (supporting).

## Supporting information

Table S1‐S4

## Data Availability

Sequences produced for this study are available in the NCBI Sequence Read Archive (BioProject: PRJNA657082, Biosample accessions: SAMN15815005‐SAMN15815012, SRA accessions: SRR12534637‐SRR12534644), and Sanger sequences from each isolate were deposited in the GenBank database with accession numbers MT893178–MT893200. Data are available from the Dryad Digital Repository: https://doi.org/10.5061/dryad.73n5tb2xv
