# Supplementary material for: Three phylogenetically distinct and culturable diazotrophs are perennial symbionts of leaf‐cutting ants
Source: Ecol Evol. 2021 Dec 14;11(24):17686–99. doi: 10.1002/ece3.8213 (PMC8717316; doi:10.1002/ece3.8213)
Supplement: Supplementary file 1 — Table S1‐S4 [file ECE3-11-17686-s001.docx]

**Appendix A**

**TABLE S1.** Core Leafcutter Bacteriome. Number and percentage of 16S rRNA sequences in bacterial genera shared by every sanitized ant sample. *Liberibacter*, *Terasakiella*, *Bartonella* and *Rhodovulum* were the main shared taxa and accounted for 98.12% of the sequences.

| **Bacterial Genus** | ***Atta laevigata*** | ***Atta sexdens*** | ***Acromyrmex***  ***rugosus*** | ***Acromyrmex***  ***coronatus*** | **Total** | | **%** |
| --- | --- | --- | --- | --- | --- | --- | --- |
| *Liberibacter*  *Terasakiella*  *Bartonella*  *Rhodovulum*  *Rhodomicrobium*  *Filomicrobium*  *Sphingopyxis*  *Rhizobium*  *Blastochloris*  *Candidatus Phycorickettsia*  *Wolbachia*  *Hyphomicrobium*  *Orientia*  *Pseudomonas*  *Zymomonas*  *Sinorhizobium*  *Acinetobacter*  *Nitratireductor*  *Agrobacterium*  *Devosia*  *Sphingomonas*  *Klebsiella*  *Methylobacterium*  *Octadecabacter*  *Massilia*  *Tistrella*  *Hirschia*  *Thalassospira*  *Chelativorans*  *Bacillus <*firmicute*s>*  *Mycobacterium*  *Halocynthiibacter*  *Gemmobacter*  *Ehrlichia*  *Maricaulis*  *Brevundimonas*  *Clostridium*  *Neorhizobium*  *Rhodopseudomonas*  *Methylocella*  *Sphingobium*  *Phenylobacterium*  *Rickettsia*  *Asticcacaulis*  *Desulfurella*  *Staphylococcus*  *Lactobacillus*  *Haemophilus*  *Candidatus Nucleicultrix*  *Candidatus Fokinia*  *Aeromonas*  *Parasaccharibacter*  *Desulfobacca*  *Rhodobacter* | 96,628  563  151  1,419  364  280  169  169  112  103  15  110  69  5  39  82  27  90  40  13  7  57  13  11  1  21  11  10  1  7  9  8  6  10  6  6  1  7  2  5  2  6  2  4  2  2  1  1  1  1  2  1  2  1 | 1,229  29  76,955  968  59  99  129  55  41  91  16  53  95  191  68  14  38  12  43  27  54  6  18  21  15  11  9  6  1  4  3  11  8  7  5  11  2  5  10  2  6  4  3  5  1  3  3  4  1  4  1  1  1  1 | 3,396  29,254  44  2,411  85  73  86  42  54  41  2  28  39  9  25  14  9  1  8  2  3  2  5  6  1  5  3  5  1  8  2  2  3  4  3  1  8  1  2  4  2  1  1  1  1  3  2  2  1  1  1  1  1  2 | 5,708  56,345  151  2,408  180  147  101  105  101  66  263  72  52  16  37  53  35  1  12  53  19  16  26  20  35  2  10  10  24  6  10  3  6  1  7  3  5  3  2  4  3  2  6  2  6  2  3  1  4  1  2  2  1  1 | 106,961  86,191  77,301  7,206  688  599  485  371  308  301  296  263  255  221  169  163  109  104  103  95  83  81  62  58  52  39  33  31  27  25  24  24  23  22  21  21  16  16  16  15  13  13  12  12  10  10  9  8  7  7  6  5  5  5 | 37.80  30.45  27.32  2.55  0.24  0.21  0.17  0.13  0.11  0.11  0.10  0.09  0.09  0.08  0.06  0.06  0.04  0.04  0.04  0.03  0.03  0.03  0.02  0.02  0.02  0.01  0.01  0.01  0.01  0.01  0.01  0.01  0.01  0.01  0.01  0.01  0.01  0.01  0.01  0.01  0.005  0.005  0.004  0.004  0.004  0.004  0.003  0.003  0.002  0.002  0.002  0.002  0.002  0.002 | |
| Total | 100,675 | 80,459 | 35,712 | 66,154 | 283,000 | | 100% |

**TABLE S2.** Core Leafcutter Bacteriome. Number and percentage of 16S rRNA sequences in bacterial genera shared by every unsanitized ant species. *Liberibacter*, *Terasakiella*, *Bartonella* and *Rhodovulum* were the main shared taxa and accounted for 97.33% of the sequences.

| **Bacterial Genus** | ***Atta laevigata*** | ***Atta sexdens*** | ***Acromyrmex***  ***rugosus*** | ***Acromyrmex***  ***coronatus*** | **Total** | **%** |
| --- | --- | --- | --- | --- | --- | --- |
| *Liberibacter*  *Bartonella*  *Terasakiella*  *Rhodovulum*  *Wolbachia*  *Nocardioides*  *Rhodomicrobium*  *Filomicrobium*  *Bacillus <*Firmicutes*>*  *Sphingopyxis*  *Rhizobium*  *Corynebacterium*  *Hyphomicrobium*  *Blastochloris*  *Orientia*  *Candidatus Phycorickettsia*  *Sphingomonas*  *Zimomonas*  *Sinorhizobium*  *Pseudomonas*  *Agromyces*  *Mycobacterium*  *Agrobacterium*  *Acinetobacter*  *Christensenella*  *Streptococcus*  *Microlunatus*  *Tessaracoccus*  *Methylobacterium*  *Niastella*  *Negativicoccus*  *Pseudonocardia*  *Bruvella*  *Devosia*  *Cutibacterium*  *Ornithinimicrobium*  *Octadecabacter*  *Streptomyces*  *Flavisolibacter*  *Rickettsia*  *Massilia*  *Moraxella*  *Staphylococcus*  *Microbacterium*  *Klebsiella*  *Hirschia*  *Ketogulonicigenium*  *Lactobacillus*  *Thalassospira*  *Synechococcus*  *Clostridium*  *Flavonifractor*  *Halocynthiibacter*  *Candidatus puniceispirillum*  *Bosea <alphaproteobacteria>*  *Candidatus nucleicultrix*  *Ehrlicha*  *Gemmobacter*  *Pseudolabrys*  *Asticcacaulis*  *Methilocella*  *Roseitalea*  *Erythrobacter*  *Rhodopseudomonas*  *Brevibacillus*  *Desulfurella*  *Pararthodospirillum*  *Alteromonas*  *Methyloceanibacter* | 113,583  155  490  996  4  38  367  272  466  115  238  253  122  129  61  88  42  36  73  42  1  12  13  36  3  4  1  31  9  4  2  11  37  16  13  20  17  2  23  1  30  24  3  1  2  9  2  6  14  1  5  5  6  3  2  6  7  3  7  4  4  2  3  2  1  5  1  1  1 | 1,371  100,022  67  1,943  101  46  67  114  43  136  42  7  69  33  126  87  87  64  17  10  3  82  68  46  77  76  2  1  52  11  20  33  3  25  30  19  14  9  10  33  2  1  19  5  19  7  15  3  5  10  5  2  5  1  3  4  3  6  2  5  4  1  2  3  3  1  1  1  1 | 10,438  11,516  38,815  2,782  944  862  140  114  4  81  95  42  58  68  63  45  12  34  28  5  110  14  17  9  5  8  34  51  11  68  8  22  15  7  3  7  7  30  6  1  3  3  8  16  7  10  5  6  2  1  4  4  4  6  10  1  2  2  2  1  1  1  3  2  1  1  2  2  1 | 3,444  34  31,416  2,328  12  44  119  70  5  87  33  4  42  59  36  40  26  21  20  81  17  3  13  7  13  3  54  3  13  2  43  5  16  9  3  2  4  1  2  5  1  6  4  8  2  3  3  9  1  8  4  7  2  6  1  4  2  2  1  2  2  6  1  1  3  1  3  1  1 | 128,836  111,727  70,788  8,049  1,061  990  693  570  518  419  408  306  291  289  286  260  167  155  138  138  131  111  111  98  98  91  91  86  85  85  73  71  71  57  49  48  42  42  41  40  36  34  34  30  30  29  25  24  22  20  18  18  17  16  16  15  14  13  12  12  11  10  9  8  8  8  7  5  4 | 39.26  34.05  21.57  2.45  0.32  0.30  0.21  0.18  0.16  0.13  0.12  0.09  0.09  0.09  0.09  0.08  0.05  0.05  0.04  0.04  0.04  0.03  0.03  0.03  0.03  0.03  0.03  0.03  0.03  0.03  0.02  0.02  0.02  0.02  0.01  0.01  0.01  0.01  0.01  0.01  0.01  0.01  0.01  0.01  0.01  0.01  0.01  0.01  0.01  0.01  0.01  0.01  0.01  0.005  0.005  0.005  0.004  0.004  0.004  0.004  0.003  0.003  0.003  0.002  0.002  0.002  0.002  0.002  0.001 |
| Total | 117,986 | 105,205 | 66,690 | 38,234 | 328,115 | 100% |

**TABLE S3.** Number of isolates in each of the bacterial genera associated with ant species. (RC) = Rio Claro and (IT) = Itirapina.

| ***Microorganism*** | **Amount of isolates** | **Ant species** |
| --- | --- | --- |
| *Methylobacterium* | 4 | *Atta laevigata* (RC)  *Atta laevigata* (IT)  *Atta sexdens* |
| *Ralstonia* | 6 | *Atta laevigata* (RC)  *Atta laevigata* (IT)  *Atta sexdens*  *Acromyrmex coronatus* |
| Burkholderiales | 6 | *Atta laevigata* (RC)  *Atta laevigata* (IT) |
| Actinomycetales | 2 | *Atta laevigata* (RC) |
| *Brachybacterium* | 1 | *Atta laevigata* (RC) |
| *Acinetobacter* | 8 | *Atta laevigata* (RC)  *Atta laevigata* (IT)  *Atta sexdens*  *Acromyrmex coronatus*  *Acromyrmex rugosus* |
| Gamaproteobacteria | 3 | *Atta sexdens*  *Acromyrmex rugosus* |
| *Pseudomonas* | 2 | *Acromyrmex rugosus* |
| Proteobacteria | 10 | *Atta laevigata* (RC)  *Atta laevigata* (IT)  *Atta sexdens* |
| **Total** | **42** |  |

**TABLE S4.** Medium used in culture tests.

| **Reagent** | **Quantities** |
| --- | --- |
| **DIGS** |  |
| Glucose | 2 g/L |
| Malic Acid | 2 g/L |
| Bacteriological Peptone | 1.5 g/L |
| Yeast Extract | 2 g/L |
| K_2_HPO_4_ | 0.5 g/L |
| MgSO_4_ .7H_2_O | 0.5 g/L |
| Glutamic Acid | 1.5 g/L |
| Agar | 15 g/L |
| Adjust pH to 6.0 with KOH or H_2_SO_4_ | |
|  | |
| **NFb** | |
| Malic acid | 5 g/L |
| K_2_HPO_4_ | 0.5 g/L |
| MgSO_4_ .7H_2_O | 0.2 g/L |
| NaCl | 0,1 g/L |
| CaCl_2_. 2H_2_O | 0.02 g/L |
| KOH | 4.5 g/L |
| Bromothymol blue solution (0.5% in 0.2 KOH) | 2 mL/L |
| FeEDTA solution (1.64% solution) | 4 mL/L |
| Vitamin solution | 1 mL/L |
| Micronutrient solution | 2 mL/L |
| Adjust pH to 6.8 with NaOH | |
|  | |
| **YMA pH 6.8** | |
| Yeast Extract | 1.0 g/L |
| Mannitol | 10 g/L |
| K_2_HPO_4_ | 0.5 g/L |
| NaCl | 0.2 g/L |
| CaCO_3_ | 1 g/L |
| Agar | 15 g/L |
|  | |
| **Micronutrient solution** | |
| CuSO_4_ . 5H_2_O | 0.04 g/L |
| ZnSO_4_ . 7H_2_O | 1.2 g/L |
| H_3_BO_3_ | 1.4 g/L |
| Na_2_MoO_4_ . 2H_2_O | 1.0 g/L |
| MnSO_4_ . H_2_O | 1.175 g/L |
|  | |
| **Vitamin solution** | |
| Biotin | 10 mg/100mL H_2_O |
| Pyridoxol HCl | 20 mg/100mL H_2_O |
